# Modified Hilbert Curve for Rectangles and Cuboids and Its Application in Entropy Coding for Image and Video Compression

**DOI:** 10.3390/e23070836

**Published:** 2021-06-29

**Authors:** Yibiao Rong, Xia Zhang, Jianyu Lin

**Affiliations:** 1 Department of Electronic Engineering, Shantou University, Shantou 515063, China; ybrong@stu.edu.cn (Y.R.); 18xzhang8@stu.edu.cn (X.Z.); 2 Guangdong Provincial Key Laboratory of Digital Signal and Image Processing, Shantou University, Shantou 515063, China; 3 Key Laboratory of Intelligent Manufacturing Technology, Shantou University, Ministry of Education, Shantou 515063, China

**Keywords:** scan route, Hilbert curve, run-length-based entropy coding, image and video compression

## Abstract

In our previous work, by combining the Hilbert scan with the symbol grouping method, efficient run-length-based entropy coding was developed, and high-efficiency image compression algorithms based on the entropy coding were obtained. However, the 2-D Hilbert curves, which are a critical part of the above-mentioned entropy coding, are defined on squares with the side length being the powers of 2, i.e., 2*^n^*, while a subband is normally a rectangle of arbitrary sizes. It is not straightforward to modify the Hilbert curve from squares of side lengths of 2*^n^* to an arbitrary rectangle. In this short article, we provide the details of constructing the modified 2-D Hilbert curve of arbitrary rectangle sizes. Furthermore, we extend the method from a 2-D rectangle to a 3-D cuboid. The 3-D modified Hilbert curves are used in a novel 3-D transform video compression algorithm that employs the run-length-based entropy coding. Additionally, the modified 2-D and 3-D Hilbert curves introduced in this short article could be useful for some unknown applications in the future.

## 1. Introduction

Entropy coding plays a critical role in data compression, such as image and video compression, etc. Two commonly used algorithms for entropy coding are Huffman coding [1] and arithmetic coding [2]. In terms of compression efficiency, arithmetic coding is preferred. However, arithmetic coding has a higher computational complexity because it requires multiplication and division during the coding process. To resolve the complexity issue, approximations are used in binary arithmetic coding algorithms, such as [3,4,5], etc. These binary arithmetic coding algorithms are practical algorithms because the coding of a multiple symbol source can always be converted to coding of a sequence of binary symbol sources. For example, in image compression, the bit-plane coding method [6] and the symbol grouping coding method [7,8] eventually convert the quantized coefficients to binaries to code.

Although the existing binary arithmetic coding algorithms solved the computational complexity issue, it is still not computationally efficient in extremely low entropy conditions because arithmetic coding algorithms encode symbols one by one. For example, to code a binary source with the probabilities p=0.999 for the symbol “0” and 1−p=0.001 for the symbol “1”, arithmetic coding needs to code 999 “0”s on average before it codes a “1”. On the other hand, run-length-based entropy coding [7,8] is much more computationally efficient for low-entropy coding situations, as it does not need to code the “0”s one by one. Note, low-entropy sources are very common in compression. For example, in subband image compression, most of the quantized coefficients in a subband are zeros. The positions of the non-zero coefficients in a subband normally form an extremely low-entropy source.

For non-stationary binary sources, the binary arithmetic coding algorithms use probability estimators to adapt to the probability variations; whereas the run-length-based entropy coding uses the symbol grouping method to handle non-stationary binary sources [7]. For coding 2-dimensional (and higher-dimensional) subband coefficient arrays, binary arithmetic coding can estimate the probabilities from the coded adjacent coefficients in different spatial directions (context modeling). However, for the run-length-based binary entropy coding, the 2-D coefficient array needs to be scanned into a 1-D array before the run-length coding can be performed. As a result, for run-length-based binary entropy coding, exploiting probability estimations in different spatial directions on the 2-D array before scanning is very difficult. Thus, to achieving coding efficiency, variations in the original 2-D signal need to be maximumly kept into the scanned 1-D array, which requires that nearby elements in the 2-D array are still nearby in the 1-D scanned array. Ideally, adjacent elements in the 2-D array are required to be adjacent elements in the 1-D scanned array, which is impossible, as can be easily shown. However, different scan routes lead to different scatterings of the 2-D nearby elements. Thus, scan routes with small scattering are desired. The Hilbert curve [9,10] is such a route.

**Figure 1:**
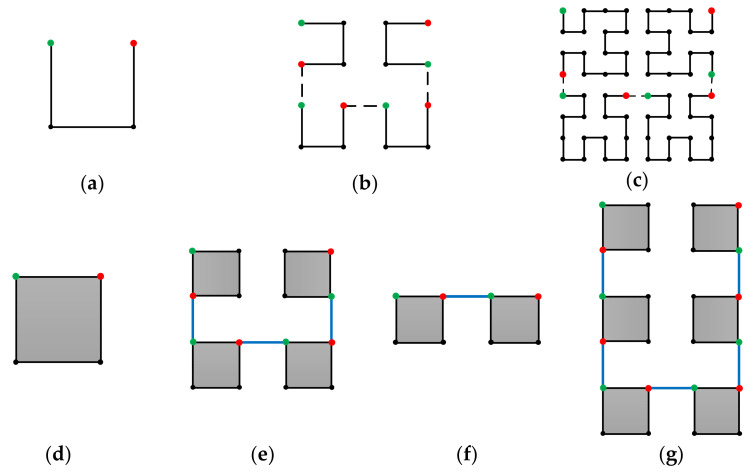
2-D Hilbert curve properties. (**a**–**c**) are, respectively, the 1st order, the 2nd order, and the 3rd order Hilbert curves. (**d**) A simple notation to represent the $i^{\mathrm{th}}$ order 2-D Hilbert curve. (**e**) Construction of the ${\left(i+1\right)}^{\mathrm{th}}$ order Hilbert curve from the $i^{\mathrm{th}}$ order Hilbert curve. (**f**) Reduction of $2^{i}$ points on the height H for the ${\left(i+1\right)}^{\mathrm{th}}$ order Hilbert curve. (**g**) Increasing of $2^{i}$ points on the height H for the ${\left(i+1\right)}^{\mathrm{th}}$ order Hilbert curve.

Figure 1 shows a 2-D Hilbert curve. As can be seen, the Hilbert curve tries to keep the $2^{k}\times 2^{k}\;\left(k=1,\;2,\;\ldots \right)$ elements in the 2-D array together in the scanned 1-D array. In fact, the locality-preserving feature of the Hilbert curve has been extensively studied [10,11,12,13]. Thus, using the Hilbert curve scan route, the variations within a 2-D subband coefficient array are greatly kept into the 1-D scanned coefficient array. Indeed, combining the Hilbert scan with the symbol grouping method, an efficient entropy coding was achieved, and high-efficiency image compression algorithms were obtained [7,8]. However, 2-D Hilbert curves are defined on a square of sizes $2^{i}\times 2^{i}\;\left(i=1,\;2,\;\ldots \right)$ [9,14]. In other words, not only the array shape is a square, but also the side length of the square can only be the powers of 2, i.e., $2^{i}$. Yet, a subband is normally a rectangle of arbitrary sizes. It is not straightforward to modify the Hilbert curve from the $2^{i}\times 2^{i}$ squares to an arbitrary rectangle. In [7,8], details of this modification are not provided.

In this short article, we provide the details of constructing the modified 2-D Hilbert curve of arbitrary rectangle sizes. Furthermore, we extend the method from a 2-D rectangle to a 3-D cuboid. The entropy coding in the 3-D transform video compression algorithm introduced in [15] uses the 3-D Hilbert curve. Test results show that the algorithm is promising. However, the original 3-D Hilbert curve is defined on a cube of side length of power of 2, i.e., size $2^{i}\times 2^{i}\times 2^{i}\;\left(i=1,\;2,\;\ldots \right)$ [16]. Because the 3-D modified Hilbert curves for cuboids were not available at the time, videos were cropped to the size of 1024×1024 for testing the algorithm prototype in [15]. The extension from a 2-D rectangle to a 3-D cuboid makes the prototype proposed in [15] a practical video compression algorithm that accommodates arbitrary rectangle video sizes. Further, Hilbert curves have been widely used in many applications, such as image data encryption, query, and retrieval [17,18], etc. Extending the original Hilbert curves to arbitrary size rectangles and cuboids could be useful for some unknown applications in the future.

## 2. Two- and Three-Dimensional Modified Hilbert Curves

### 2.1. The 2-D Modified Hilbert Curve

The original 2-D Hilbert curve connects a square array of the size of $2^{i}\times 2^{i}$. We denote it as the $i^{th}$ order Hilbert curve. Hilbert curves of orders i=1, i=2, and i=3 are respectively shown in Figure 1a–c. There is an important property of the Hilbert curve. As can be seen from Figure 1a–c, the starting and the ending points (green and red points in the graphs) are always on one side of the $2^{i}\times 2^{i}$ square. Since the starting and the ending points are at the ends of one side of the Hilbert curve square, a Hilbert curve of any order i can easily be represented by a square with labeled starting and ending points like Figure 1d, when the internal structure does not need to be shown.

Now, we can show that an ${\left(i+1\right)}^{\mathrm{th}}$ order Hilbert curve can be easily constructed from four $i^{\mathrm{th}}$ order Hilbert curves. First, replace the 4 points in the 1^st^ order Hilbert curve in Figure 1a with four $i^{\mathrm{th}}$ order Hilbert curves whose starting and ending points are arranged as indicated in Figure 1e; then, connect the ending points and starting points of the $i^{\mathrm{th}}$ Hilbert curves orderly as indicated in Figure 1e, and the ${\left(i+1\right)}^{\mathrm{th}}$ order Hilbert curve is constructed. With the 1^st^ order Hilbert curve provided by Figure 1a and the method of constructing the ${\left(i+1\right)}^{\mathrm{th}}$ order Hilbert curve from the $i^{\mathrm{th}}$ Hilbert curve, we can construct the Hilbert curve of any order by mathematical induction.

Now, our task is to construct a scanning route that is close to the Hilbert curve for a 2-D array of size W×H, where *W* and *H* are the element numbers along the vertical and horizontal direction, respectively.

**Figure 2:**
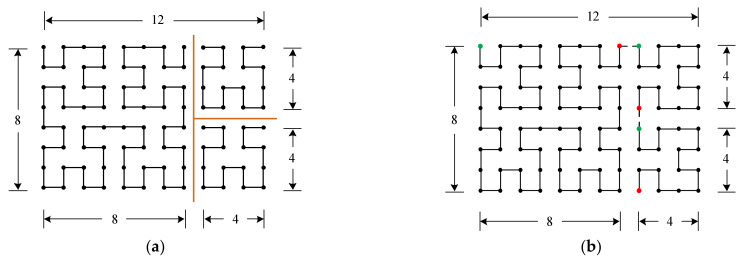
Construction of the 2-D modified Hilbert curve for the array size 12 × 8. (**a**) Three Hilbert curves are constructed for the 3 divided $2^{i_{n}}\times 2^{i_{n}}$ sub-square arrays. (**b**) By selecting appropriate directions for each of the 3 Hilbert curves and connecting the 3 Hilbert curves, the modified 2-D Hilbert curve is constructed.

The basic idea is to divide the W×H rectangle array into N small square arrays of the size $2^{i_{n}}\times 2^{i_{n}},\;n=1,\;2,\;\ldots ,N$, and $i_{1}\geq i_{2}\geq \ldots \geq i_{N}$. For example, a 12×8 rectangle array can be divided into three $2^{i_{n}}\times 2^{i_{n}}$ square arrays with $i_{1}=3$ and $i_{2}=i_{3}=2$. Apparently, within each square, an ${\left(i_{n}\right)}^{\mathrm{th}}$ order Hilbert curve can be easily constructed as shown in Figure 2a. By appropriately arranging the directions of each Hilbert curve, the 3 Hilbert curves are connected to form the desired route, as shown in Figure 2b.

To form a route close to the Hilbert curve, there are two requirements. First, the largest $i_{n}$ needs to be selected in order, i.e., select the largest $i_{1}$ first, then the largest $i_{2}$, …, etc. Without this restriction, the constructed curves may deviate from the Hilbert curve significantly. As an extreme example, for the 12×8=96 points in Figure 2, one can simply choose $i_{1}=i_{2}=\ldots =i_{96}=0$, i.e., use the 96 points as 96 small square arrays. In this case, there are too many possible routes. Most of them are not close to the Hilbert curve at all, for example, the raster scan. Secondly, the ending point of the Hilbert curve in the $n^{\mathrm{th}}$ square must be adjacent to the starting point of the Hilbert curve of the ${\left(n+1\right)}^{\mathrm{th}}$ square, like the example shown in Figure 2b. For design convenience, the first requirement may not be satisfied strictly sometimes. However, the second requirement must be satisfied.

**Figure 3:**
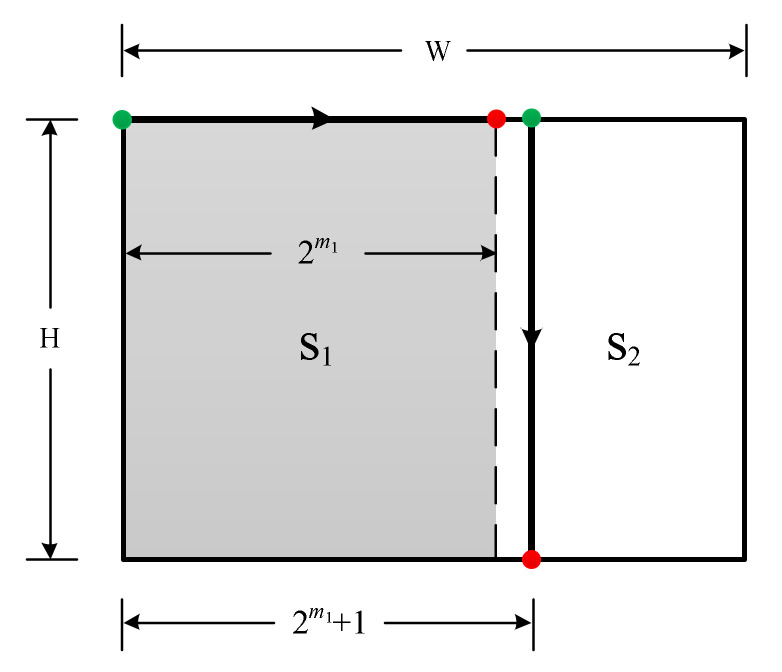
Illustration of the 2-D Modified Hilbert curve construction procedures on a W×H rectangle array, where W≥H.

Based on the above observations, the procedures of our design method are provided as follows (note, the method is not unique). Without loss of generality, we consider rectangle arrays with W≥H:

1. Find integer $m_{1}$ such that $2\times 2^{m_{1}}>W$ and $2^{m_{1}}\leq W$.
2. Construct a modified Hilbert curve within the sub-rectangle S_1_ of size $2^{m_{1}}\times H$. S_1_ is a special rectangle with the width being $2^{m_{1}}$. The starting and the ending points of S_1_ need to be at the ends of the S_1_’s top width, as indicated in Figure 3. The construction details for this step is provided shortly. (Note, we use “width” and “height” to represent the number of elements in the two orthogonal directions of a rectangle array throughout the paper. They are not the geometric lengths. Do not get confused with the illustrating diagrams used in the paper.)
3. Once the modified Hilbert curve for the rectangle array S_1_ is constructed, the construction of the remaining rectangle array S_2_ goes back to step (1) with the starting and the ending points indicated in Figure 3. However, the array size of S_2_ is less than half of the original W×H rectangle.
4. By iterating steps 1 to 3, the subsequent remaining S_2_’s become smaller and smaller very quickly, with speed faster than $0.5^{k}$, where k is the iteration number. The iteration stops when the remaining S_2_ is of size $2^{l}\times H^{\prime }$, and the construction is complete. Note, a size of $2^{l}\times H^{\prime }$ can always be achieved because the smallest $H^{\prime }$ can be 1.

Note, when iterating the three steps 1 to 3, if for S_2_, the height H is larger than $W-2^{m_{1}}$ like the situation shown in Figure 3, then H plays the role of the width W for the new iteration on S_2_ because we assumed the initial condition of W≥H for each iteration. In this case, the design route changes direction because it is always along the width direction, see Figure 3. If for S_2_ the height H is still smaller than $W-2^{m_{1}}$, the iteration continues along the same design route direction. In the following example, we show a specific design to provide a more intuitive understanding of the procedures.

**Figure 4:**
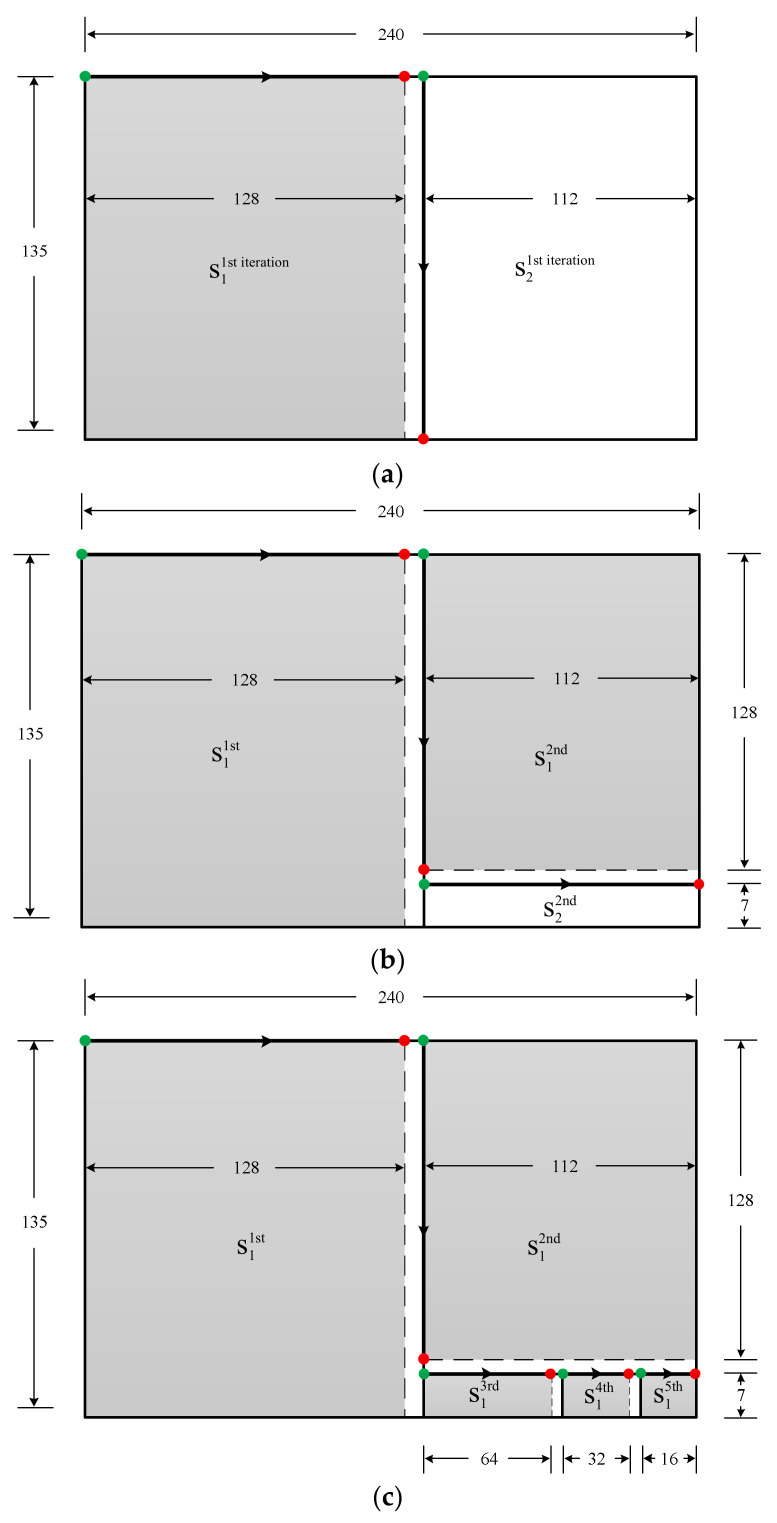
The example of designing the 2-D modified Hilbert curve for the 240 × 135 array: (**a**) the 1st iteration, (**b**) the 2nd iteration, and (**c**) the 3rd, 4th, and the 5th iteration.

Suppose we want to design a modified 2-D Hilbert curve for a practical size of W=1920/8=240 and H=1080/8=135, which is the subband size from the 8×8 subband decomposition on a 1920×1080 image, the standard HDTV size. Following the design procedures:

Iteration 1: $m_{1}=7$, and sub-rectangle arrays S_1_ and S_2_ for the 1st iteration are: $S_{1}^{1\mathrm{st}\;\mathrm{iteration}}=128\times 135$, $S_{2}^{1\mathrm{st}\;\mathrm{iteration}}=112\times 135$, as shown in Figure 4a.

Iteration 2: Because $H=135>112=W-2^{m_{1}}$, the 135 side of $S_{2}^{1\mathrm{st}\;\mathrm{iteration}}$ needs to be the width for the 2nd iteration. Then, for the second iteration on the 112×135 array, $m_{2}=7$, the starting (green) point and the ending (red) point are aligned vertically, changing the design route direction, see Figure 4b. The resulting sub-rectangle arrays S_1_ and S_2_ for the 2nd iteration are $S_{1}^{2\mathrm{nd}}=112\times 128$, $S_{2}^{2\mathrm{nd}}=112\times 7$, as shown in Figure 4b.

Iteration 3: Because $112>7=135-2^{m_{2}}$, the 112 side of $S_{2}^{2\mathrm{nd}}$ needs to be the width in the 3^rd^ iteration. Then, for the 3^rd^ iteration on the 112×7 array, $m_{3}=6$, and the starting (green) point and the ending (red) point are aligned horizontally, changing the design route direction again, see Figure 4c. The resulting sub-rectangles S_1_ and S_2_ for the 3^rd^ iteration are: $S_{1}^{3\mathrm{rd}}=64\times 7$, $S_{2}^{3\mathrm{rd}}=48\times 7$, see Figure 4c. Note, $S_{2}^{3\mathrm{rd}}=S_{1}^{4\mathrm{th}}+S_{1}^{5\mathrm{th}}$ in Figure 4c.

Iteration 4: Because $7<48=112-2^{m_{3}}$, the 48 side of $S_{2}^{3\mathrm{rd}}$ is still the width for the 4^th^ iteration. Thus, for the 4^th^ iteration on $S_{2}^{3\mathrm{rd}}=48\times 7$, $m_{4}=5$, the starting (green) point and the ending (red) point are still aligned horizontally, with no design route direction change. The resulting sub-rectangles S_1_ and S_2_ for the 4^th^ iteration are: $S_{1}^{4\mathrm{th}}=32\times 7$, $S_{2}^{4\mathrm{th}}=16\times 7$, see Figure 4c. Note, $S_{2}^{4\mathrm{th}}=S_{1}^{5\mathrm{th}}$ in Figure 4c.

Iteration 5: Similar to Iteration 4, no change of the design route direction is needed. However, the width for the 5^th^ iteration is $16=2^{4}$. By selecting $m_{4}=4$, $S_{1}^{5\mathrm{th}}=16\times 7$, and $S_{2}^{5\mathrm{th}}=0\times 7$, the design completes as indicated in Figure 4c.

Now we go back to provide the details of step 2 of the design procedures. In other words, we need to design a modified Hilbert curve for a rectangle with the width being $2^{m_{1}}$ and the height being H. There are three possible situations, (A) $H<2^{m_{1}}$, (B) $H>2^{m_{1}}$, and (C) $H=2^{m_{1}}$. Condition (C) is trivial, where the sub-rectangle is a $2^{m_{1}}\times 2^{m_{1}}$ square, and the construction is simply the original Hilbert curve.

**Condition (A)** $H<2^{m_{1}}$:

We start from the ${\left(m_{1}\right)}^{\mathrm{th}}$ order Hilbert curve, whose height is $2^{m_{1}}$. Thus, we need to reduce the height by $\Delta H=2^{m_{1}}-H$. Recall that an integer B can be converted into its binary format $b_{s}b_{s-1}\ldots b_{1}b_{0}$, i.e., B can be decomposed as

(1) $$B=b_{s}2^{s}+b_{s-1}2^{s-1}+\ldots +b_{1}2^{1}+b_{0}2^{0},$$

where the $b_{i}$’s are either 0 or 1. Decomposing ΔH using (1), we can perform a reduction of ΔH step by step, with each step achieving a reduction of $2^{i}$ points. For example, suppose ΔH=13. Then, from (1), we have ΔH=8+4+1, i.e., $b_{3}=1$, $b_{2}=1$, $b_{1}=0$, and $b_{0}=1$. Thus, we need to reduce 8 points, 4 points, and 1 point on H to achieve the total reduction of ΔH=13 points.

To perform a reduction of $2^{i}$ points, first, a reduction of $2^{i}$ points on the ${\left(i+1\right)}^{\mathrm{th}}$ order Hilbert curve is straightforward. By inspection, it can be seen that the top two sub-squares in Figure 1e can be removed, leading to Figure 1f. Then, a reduction of $2^{i}$ points on the ${\left(i+1\right)}^{\mathrm{th}}$ order Hilbert curve is achieved.

**Figure 5:**
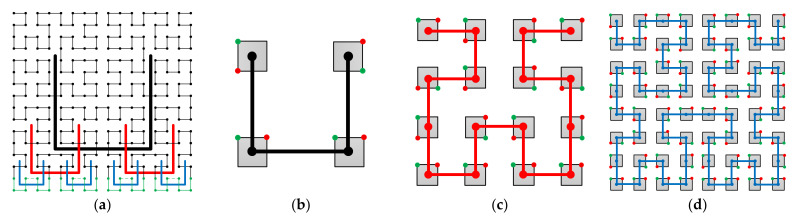
(**a**) The 4^th^ order 2-D Hilbert curve; The 4^th^ order 2-D Hilbert curve represented using (**b**) four 3^rd^ order sub-curves; (**c**) sixteen 2^nd^ order sub-curves; and (**d**) sixty-four 1^st^ order sub-curves.

Next, we observe the following property: For an opening-toward-top Hilbert curve of any order, the bottom sub-Hilbert curves always have the same opening-toward-top orientation. As an example, in Figure 5a, a 4^th^ order opening-toward-top Hilbert curve is plotted. The 4^th^ order Hilbert curve can be represented using the structure of Figure 5b, i.e., the main structure is an opening-toward-top 1^st^ order Hilbert curve with 4 sub-curves, which are four 3^rd^ order Hilbert curves denoted by four small shaded squares. As just described above, the removal of the top two sub-squares, or, equivalently, a reduction of 8 points in H in this case, can be easily achieved. Now, it is important to observe from Figure 5b that the bottom two shaded squares, i.e., the bottom two 3^rd^ order sub-Hilbert curves, are also opening-toward-top Hilbert curves. When the original 4^th^ order Hilbert curve is represented using the structure of Figure 5c, for each of the bottom two opening-toward-up 3rd order sub-Hilbert curves, the removal of the top two sub-squares, or equivalently a reduction of 4 points on H in this case, can be achieved. Similarly, it can be seen from Figure 5a,d that reductions of 2 points and 1 point on H can be achieved.

**Figure 6:**
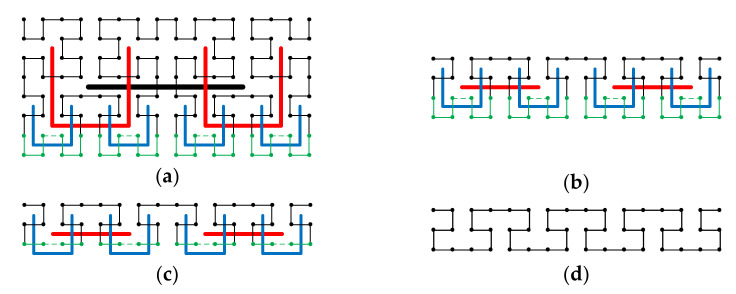
The specific example of reducing H from 16 to 3, i.e., ΔH=13. (**a**) The modified curve by an 8-point reduction on H of the 4th order Hilbert curve shown in Figure 5a; (**b**) a further reduction of 4 points on (**a**); (**c**) a further reduction of 1 point on (**b**); (**d**) the final result of a total reduction of ΔH=13 points on the 4th order Hilbert curve is achieved.

Figure 6 shows a specific example of how the 4^th^ order Hilbert curve is reduced by ΔH=13=8+4+1. In Figure 5a, the original 4^th^ order H=16 Hilbert curve is shown. Reductions on H by 8, 4, and 1 in each step are respectively shown in Figure 6a–c. After all the sub-reductions are completed, the total reduction of ΔH=13 is achieved in Figure 6d.

**Condition (B)** $H>2^{m_{1}}$:

We need to increase the height by $\Delta H=H-2^{m_{1}}$. Note, since $2\times 2^{m_{1}}>W\geq H$, $\Delta H=H-2^{m_{1}}<2\times 2^{m_{1}}-2^{m_{1}}=2^{m_{1}}<H$, i.e., ΔH<H. Thus, similar to condition (A), ΔH is decomposed by (1), and we can increase H by $2^{i}$ points in each step because the increase in height by $2^{i}$ points on the ${\left(i+1\right)}^{\mathrm{th}}$ order Hilbert curve can be achieved using the modification from Figure 1e–g.

With the height of the ${\left(m_{1}\right)}^{\mathrm{th}}$ order Hilbert curve reduced or increased to H, procedure 2 of the iterative design method described earlier is performed. The modified 2-D Hilbert curve on a W×H rectangle array using the iterative design procedures is completed.

**Figure 7:**
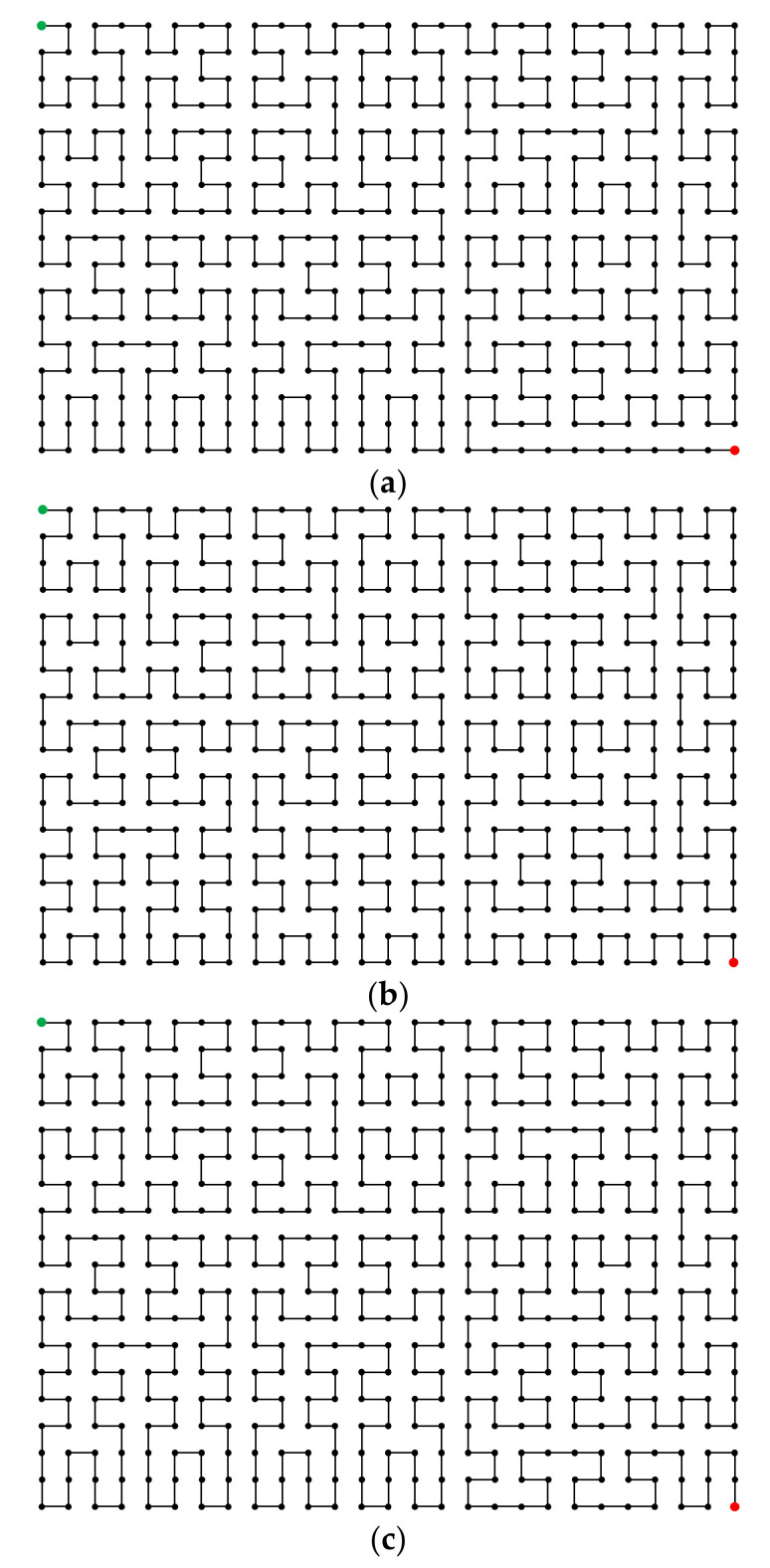
The modified 2-D Hilbert curves of sizes (**a**) 27×17, (**b**) 27×18, and (**c**) 27×19, constructed using the proposed method.

To provide more intuitions, the 2-D modified Hilbert curves of sizes 27×17, 27×18, and 27×19, constructed using the proposed method, are shown in Figure 7. In addition, the MATLAB codes implementing the proposed method are available in [19]. As can be easily checked, the algorithm runs reasonably fast, and the results can be obtained instantly.

### 2.2. The 3-D Modified Hilbert Curve

In [15], the binary run-length-based symbol grouping entropy coding method is used in video compression. For the 3-D transform video compression algorithm introduced in [15], conventional motion compensation is not used in order to improve the computational complexity. Instead, a 4-band SCWP transform [20] is performed along the time dimension. In other words, the first step of the video compression algorithm is a 3-D transform. Thus, the transformed coefficients are 3-D subband arrays.

In entropy coding of the quantized 3-D subband coefficient arrays, the 3-D Hilbert curve scan was used to maximally keep the correlations in the 3-D subband into the 1-D scanned array. Because the original 3-D Hilbert curves are for cubes of side length $2^{i}$, in [15], the 1920×1080 test videos were cropped to a size of 1024×1024 for testing. Apparently, to accommodate an arbitrary rectangle video size, the original 3-D Hilbert curve needs to be modified. Below, we extend the modification method introduced in Section 2.1 for 2-D arrays to 3-D conditions. The 3-D arrays are of size W×H×D, where W and H are the width and height of the cuboid array, D is the third dimension denoting the depth here, which corresponds to the time dimension of the input video.

The depth D of the 3-D decomposed subband is determined by the parameter “group of pictures” (GOP), which is normally selected to be the powers of 2. As a result, the depths D are also the powers of 2, i.e., $D=2^{d},\;d=0,\;1,\;2,\;\ldots$ For example, the GOP used in [15] was 32, which leads to the depths D of the 3-D subbands being 8, 4, 2, or 1 (for details, please refer to [15]). Furthermore, compared with W and H, D is normally much smaller in our case.

**Figure 8:**
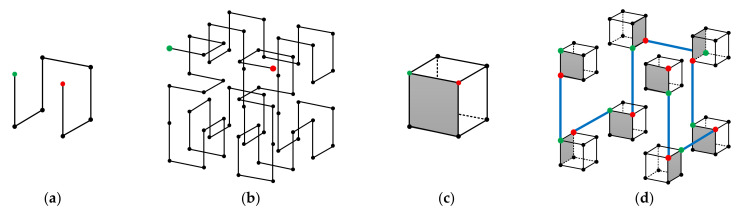
3-D Hilbert curve properties. (**a**,**b**) are respectively the 1st order and the 2nd order 3-D Hilbert curves. (**c**) A simple notation to represent the $i^{\mathrm{th}}$ order 3-D Hilbert curve. (**d**) Construction of the ${\left(i+1\right)}^{\mathrm{th}}$ order Hilbert curve from the $i^{\mathrm{th}}$ order Hilbert curve.

We begin from the original 3-D Hilbert curve. Again, we denote a 3-D Hilbert curve of size $2^{i}\times 2^{i}\times 2^{i}$, the $i^{\mathrm{th}}$ order 3-D Hilbert curve. Figure 8a,b, respectively, show the 1^st^ order and the 2^nd^ order 3-D Hilbert curve. Similar to the 2-D situation, the starting and the ending points of any order 3-D Hilbert curves are at the two ends of one side of the cube. Therefore, as shown in Figure 8c, when the internal structure is not needed, a 3-D Hilbert curve of any order i can be represented by a cube with the starting and ending points labeled. With this simple and intuitive representation, the construction of the ${\left(i+1\right)}^{\mathrm{th}}$ order 3-D Hilbert curve from the $i^{\mathrm{th}}$ order 3-D Hilbert curve can be easily demonstrated by Figure 8d. By mathematical induction, given (1) the 1^st^ order 3-D Hilbert curve and (2) the method of constructing the ${\left(i+1\right)}^{\mathrm{th}}$ order 3-D Hilbert curve from the $i^{\mathrm{th}}$ order 3-D Hilbert curve, the 3-D Hilbert curve of any order can be constructed.

Without loss of generality, assume W≥H. As mentioned in our application, D is the powers of 2 and is normally much smaller than W and H. In other words, the modified 3-D Hilbert curve is of size $W\times H\times D=W\times H\times 2^{d}$, and D is much smaller than W and H. Exploiting these features, the extension to 3-D from 2-D can borrow the 2-D construction procedures introduced in Section 2.1 as follows.

**Figure 9:**
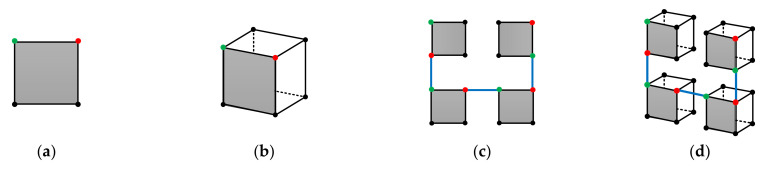
Extension of the 2-D construction to 3-D construction for size $\left(cD\right)\times \left(rD\right)\times D$, where c and r are integers. (**a**) The smallest construction block for 2-D, the D×D square. (**b**) The smallest construction block for 3-D, the D×D×D. cube. (**c**) The 2D×2D size 2-D Hilbert curve, constructed from the 2-D smallest construction block. (**d**) The 2D×2D×D size 3-D Hilbert curve, constructed from the 3-D smallest construction block borrowing the 2-D construction structure of (**c**).

First, consider the situation where W and H are multiples of D, i.e., the cuboid of size $W\times H\times D=\left(cD\right)\times \left(rD\right)\times D$, where W=cD, H=rD, c and r are integers. In this case, we can directly extend the 2-D construction method to 3-D construction. To see that, the $d^{\mathrm{th}}$ order original 2-D Hilbert curve is compared with the $d^{\mathrm{th}}$ order original 3-D Hilbert curve in Figure 9a,b. Because the D×D×D cube is the smallest construction block for the 3-D curve and the D×D square is the smallest construction block for the 2-D curve, the 3-D construction of size $W\times H\times D=\left(cD\right)\times \left(rD\right)\times D$ can directly borrow the 2-D construction structure of size $W\times H=\left(cD\right)\times \left(rD\right)$. Figure 9c,d intuitively show the 2-D to 3-D extension by comparing the 2-D Hilbert curve of size 2D×2D with the modified 3-D Hilbert curve of size 2D×2D×D.

Next, we consider the situation where W and H are not multiples of D, but W≥D. In this case, we can still borrow the 2-D construction structure, i.e., use the 2-D iterative route similar to Figure 3 and Figure 4c for the height-width (W-H) surface of the 3-D cuboid. This is similar to what we performed in Figure 9c,d, where W and H are multiples of D. The difference is that, in this case, ΔH is not a multiple of D. In this case, we need to consider the non-zero terms in $\Delta H=\sum b_{i}2^{i}$ that are smaller than D, i.e., the $2^{i}<D$ terms. The $2^{i}\geq D$ terms are multiples of D, which is the situation previously considered. The $2^{i}<D$ terms in ΔH, however, need to be handled on the 3-D cubes at the bottom. For example, if in Figure 9d we want to construct a modified 3-D Hilbert curve of size 2D×1.5D×D instead of 2D×2D×D, then the bottom cubes in Figure 9d need to be reduced by $0.5D=2^{d-1}$ points to achieve H=1.5D.

**Figure 10:**
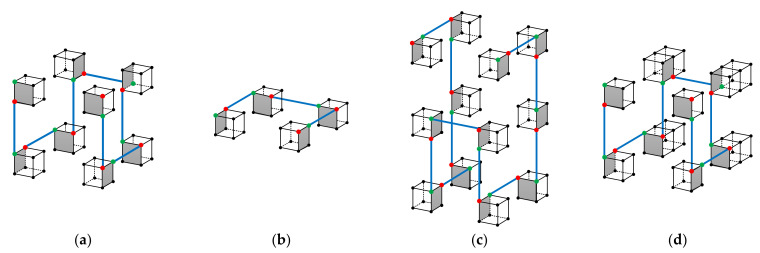
Point increasing and reducing operations on the ${\left(i+1\right)}^{\mathrm{th}}$ order 3-D Hilbert curve. (**a**) The ${\left(i+1\right)}^{\mathrm{th}}$ order 3-D Hilbert curve. (**b**) The i points are reduced on H. (**c**) The i points are increased on H. (**d**) The situation where i points need to be increased in a different direction.

Therefore, we need to consider adding or reducing $2^{i}$ points on the $D\times D\times D=2^{d}\times 2^{d}\times 2^{d}$ cube, where 0≤i≤d−1. This is not difficult. (1) Similar to the 2-D situation, observe in Figure 10a that the bottom 4 sub-cubes (sub-Hilbert curves) are of the same opening-toward-top orientation as its original 3-D Hilbert curve because they all have the starting and ending points at the top surface of the cube. (2) For the 3-D Hilbert curve of size $2^{d}\times 2^{d}\times 2^{d}$, indicated in Figure 10a, reducing and increasing $2^{d-1}$ points on H can be achieved by Figure 10b,c respectively. Combining (1) and (2) above, reducing or increasing $2^{i}\;\left(0\leq i\leq d-1\right)$ points on H can be achieved on a 3-D Hilbert curve of size $2^{d}\times 2^{d}\times 2^{d}$.

**Figure 11:**
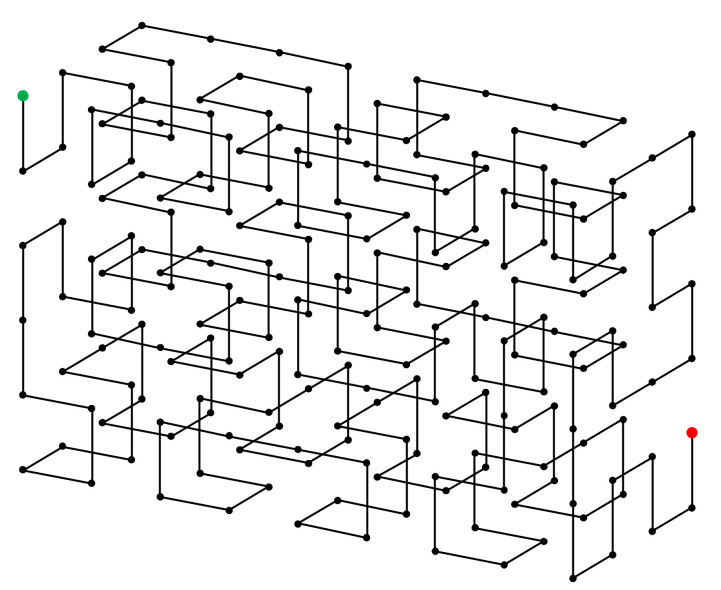
The modified 3-D Hilbert curve of size 9×6×4, constructed using the proposed method.

Finally, during the 3-D construction described above, for the height-width (W-H) surface of the 3-D cuboid, we can exploit the 2-D iterative route similar to Figure 3 and Figure 4c as long as W≥D is satisfied for the remaining S_2_’s in the W-H surface. For example, we can realize the modified 3-D Hilbert curve for the size of 240×135×8 following exactly the 2-D route shown in Figure 4c, which was used for constructing the 240×135 2-D modified Hilbert curve. Nevertheless, a construction may end up with a residue S_2_ on the W-H surface, whose height and width are both smaller than D. For example, using the above method to construct the modified 3-D Hilbert curve of the size 243×135×8, we end up with a residue cuboid of size 3×7×8. Then, the 2-D Figure 3 iterative procedure cannot proceed for the W-H surface anymore. We have to consider the situation of constructing a modified 3-D Hilbert curve of the size W×H×D with $D=2^{d}>W\geq H$.

However, in the 3-D transform video application, D is very small. As mentioned above, in [15], the maximum D=8 even if the GOP used is 32. When D=8, the maximum residue cuboid is only 7 × 7 × 8, which is very small. For such tiny residue cuboids, using some other routes, such as the raster scan, would not lead to any noticeable effect on the final video compression results. On the other hand, the design for the situation of $D=2^{d}>W\geq H$ is complex, and thus, for the application of coding the 3-D transformed coefficients in video compression, we can just use a simple scan route for the residue cuboids with D>W≥H. We implemented in MATLAB such 3-D extension with small D>W≥H residue cuboid connected using the raster scan, which is available at [19]. Figure 11 shows a modified 3-D Hilbert curve of size 9×6×4 (i.e., $D=2^{2})$ produced by MATLAB codes.

We will not lengthily go into the design on the condition $D=2^{d}>W\geq H$. For completeness, we only briefly describe that the design is possible using similar ideas we have used up to now. Note, there can be some other methods to handle the $D=2^{d}>W\geq H$ situation because the design method is not unique.

For the $D=2^{d}>W\geq H$ situation, first, consider the situation where either W or H is a power of 2. Without loss of generality, assume $H=2^{h}$. Observe that the sizes W×H×D, D×H×W, …, etc., i.e., all the 6 permutations, are the same for our curve construction task. In order to exploit our previously developed construction techniques, we need to change the roles of W, H, and D. Because D is the longest side, we need to use $D=2^{d}$ as the width. Since $H=2^{h}<D$, use H as the depth. Then, the construction finishes nicely in one step.

For the more difficult situation, where both W and H are not powers of 2, decompose the shortest side H into a sum of $2^{i}$ using equation (1): $H=2^{h_{0}}+2^{h_{1}}+\ldots +2^{h_{n}}$, where $h_{0}>h_{1}\ldots >h_{n}$ ($h_{0}$ corresponds to the most significant bit, i.e., $2^{h_{0}}>\frac{1}{2}H$). Then, the construction on the $D\times W\times 2^{h_{0}}=2^{d}\times W\times 2^{h_{0}}$ cuboid is immediately achieved as described above. To increase the thickness from $2^{h_{0}}$ to H, the point-increasing operation needs to be along the direction as illustrated in Figure 10d. For the 4 length-increased sub-cubes at the back in Figure 10d, the bottom 2 sub-cubes can use the point-increasing operation we already used, i.e., the one from Figure 10a to Figure 10c, but the top 2 sub-cubes need to use a different point-increasing structure, which is skipped here. We may also need to perform multiple point-increasing operations and then perform a point-decreasing operation to achieve the desired value H, and the operations need to be performed individually for the sub-cubes at the back of the $D\times W\times 2^{h_{0}}$ cuboid. The sizes of the sub-cubes can be different depending upon the W value. As a result, the implementation is complex. We will not go into the details further since currently, there is no immediate application.

## 3. Conclusions and the Near Future Work

We have shown the method of modifying the 2-D Hilbert curve to fit an arbitrary W×H rectangle array and the method of modifying the 3-D Hilbert curve to fit a cuboid array of size $W\times H\times 2^{d}$. These modified Hilbert curves can be used in entropy coding for image and video compression. Furthermore, since the construction of the modified 2-D and 3-D Hilbert curves is not straightforward, the methods presented in this short article could be useful for some unknown applications in the future.

The 2-D modified Hilbert curve has already been used in the run-length-based symbol grouping entropy coding method for lossy and lossless image compression. High compression efficiency is achieved, as shown in [7,8].

Because of using the 3-D Hilbert curve, the video compression algorithm prototype introduced in [15] only tested videos with the cropped size of 1024×1024, although some promising results were shown. On applying the 3-D modified Hilbert curve for coding to the 3-D subband coefficients so that the algorithm can handle arbitrary video sizes, together with some other fine tunings, we are completing the video compression algorithm very soon. We will systematically compare the performances of the new video compression algorithm with state-of-the-art video compression algorithms, such as HEVC, etc., in terms of compromise between complexity and compression efficiency. From the preliminary test results shown in [15], we expect that the final completed video compression algorithm using the 3-D modified Hilbert curve developed in this paper will be competitive to state-of-the-art video compression algorithms in certain important situations, such as the compression at the high video quality.

## References

[1] Huffman D. (1952). A method for the construction of minimum redundancy codes. Proc. Inst. Radio Eng..

[2] Witten I.H., Neal R.M., Cleary J.G. (1987). Arithmetic coding for data compression. Commun. ACM.

[3] Pennebaker W.B., Mitchell J.L., Langdon G.G., Arps R.B. (1988). An overview of the basic principles of the Q-coder adaptive binary arithmetic coder. IBM J. Res. Dev..

[4] Douglas Withers W. (2001). A Rapid Probability Estimator and Binary Arithmetic Coder. IEEE Trans. Inform..

[5] Belyaev E., Forchhammer S., Liu K. (2017). An Adaptive Multialphabet Arithmetic Coding Based on Generalized Virtual Sliding Window. IEEE Signal Process. Lett..

[6] Taubman D.S., Marcellin M.W. (2002). JPEG2000: Image Compression Fundamentals, Standards, and Practice.

[7] Lin J. (2019). A New Perspective on Improving the Lossless Compression Efficiency for Initially Acquired Images. IEEE Access.

[8] Lin J. (2020). Reversible Integer-to-Integer Wavelet Filter Design for Lossless Image Compression. IEEE Access.

[9] Wikipedia. https://en.wikipedia.org/wiki/Hilbert_curve.

[10] Hilbert D. (1891). Über die stetige Abbildung einer Linie auf ein Flächenstück. Math. Ann..

[11] Moon B., Jagadish H., Faloutsos C. (2001). Analysis of the Clustering Properties of Hilbert Space-filling Curve. IEEE Trans. Knowl. Data Eng..

[12] Jafadish H.V. (1997). Analysis of the Hilbert curve for representing two-dimensional space. Inf. Process. Lett..

[13] Abel D.J., Mark D.M. (1990). A Comparative Analysis of Some Two-Dimensional Orderings. Int. J. Geogr. Inf. Syst..

[14] Liu X., Schrack G.F. (1996). Encoding and decoding the Hilbert order. Softw. Pract. Exper..

[15] Lin J. Improving the Compression Efficiency for Transform Video Coding. Proceedings of the 2017 4th International Conference on Systems and Informatics (ICSAI).

[16] Liu X., Schrack G.F. (1997). An algorithm for encoding and decoding the 3-D Hilbert order. IEEE Trans. Image Process..

[17] Bourbakis N., Alexopoulos C. (1992). Picture data encryption using scan patterns. Pattern Recognit..

[18] Hu F.C., Tsai Y.H., Chung K.L. (2000). Space-filling approach for fast window query on compressed images. IEEE Trans. Image Process..

[19] https://stumail-my.sharepoint.cn/:u:/g/personal/jianyulin_stu_edu_cn/EZxWBMLZ9ndCiKCI-QwtzM4BxL0IS6MtaEyZ2A-LpLl3dA.

[20] Lin J., Smith M.J.T. (2013). Spectrum Decomposition for Image/Signal Coding. IEEE Trans. Signal Process..

